# The Use of a Novel Quantitative Marker of Echogenicity of Pleural Fluid in Parapneumonic Pleural Effusions

**DOI:** 10.1155/2020/1283590

**Published:** 2020-10-05

**Authors:** Charalampos Varsamas, Alexandros Kalkanis, Konstantinos I. Gourgoulianis, Foteini Malli

**Affiliations:** ^1^Respiratory Medicine Department, University of Thessaly, School of Medicine, Larissa, Greece; ^2^Louvain University Center for Sleep and Wake Disorders, Leuven, Belgium; ^3^Respiratory Disorders Lab, Nursing Department, University of Thessaly, Larissa, Greece

## Abstract

**Background:**

Thoracic ultrasound is an essential tool in the daily clinical care of pleural effusions and especially parapneumonic pleural effusions (PPEs), in terms of diagnosis, management, and follow-up. Hypoechogenicity index (HI) is a quantitative marker of pleural fluid echogenicity. We aimed to examine associations of HI with pleural inflammation in patients with PPE.

**Methods:**

All patients included underwent a thoracic ultrasound with HI determination at the first day of their admission for a PPE. Thoracentesis was performed in all patients. Demographics, laboratory measurements, and clinical data were collected prospectively and recorded in all subjects.

**Results:**

Twenty-four patients with PPE were included in the study. HI was statistically significantly correlated with intensity of inflammation as suggested by pleural fluid LDH (*p* < 0.001, *r* = −0.831), pleural fluid glucose (*p*=0.022, *r* = 0.474), and pleural fluid pH (*p* < 0.001, *r* = 0.811). HI was correlated with ADA levels (*p*=0.005, *r* = −0.552). We observed a statistically significant correlation of HI with pleural fluid total cell number (*p* < 0.001, *r* = −0.657) and polymorphonuclears percentage (*p*=0.02, *r* = −0.590), as well as days to afebrile (*p*=0.046, *r* = −0.411), duration of chest tube placement (*p* < 0.001, *r* = −0.806), and days of hospitalization (*p*=0.013, *r* = −0.501). *Discussion*. HI presents a fast, easily applicable, objective, and quantitative marker of pleural inflammation that reliably reflects the intensity of pleural inflammation and could potentially guide therapeutic management of PPE.

## 1. Introduction

Parapneumonic pleural effusion (PPE) refers to a pleural effusion which is associated with an infection of the pleural space. The majority of PPEs resolve with standard management such as antibiotics and pleural fluid drainage. Despite the evolution of therapeutic strategies, 20% of patients with pleural infection will require surgical intervention, thus prolonging the hospital stay, raising the annual costs, and deteriorating the clinical outcomes [1]. Therefore, it is important to identify those patients with PPE that will require more aggressive initial interventions in order to improve their prognosis. Clinical trials have previously been conducted with the aim to improve drainage outcome in patients with complicated PPE with promising results; however, data on early, simple, and fast markers of progression of uncomplicated to complicated PPE are currently lacking [2, 3].

Over the previous decades, thoracic ultrasound (TUS) has been included as an essential tool in the daily clinical practice in terms of diagnosis, management, and follow-up of pleural disease, including PPE [4, 5]. TUS offers not only a straightaway recognition of a pleural effusion but a potential reliable marker for the identification of fibrin and loculations. In addition, a proper spot for a successful thoracocentesis can be selected, therefore reducing the risk of pneumothorax and/or hemothorax [6, 7]. However, an objective and quantitative marker of pleural inflammation that could potentially guide therapeutic management is not widely available.

We have previously reported hypoechogenicity Index (HI) as a quantitative, objective image marker to evaluate the echogenicity of pleural effusions of various causes. The HI is a simple marker of density of pleural effusion that requires less than 1 minute for the calculation of every image taken while it reflects the summary of the pixels that a pleural effusion can generate. HI has been successfully correlated with pleural fluid biomarkers such as pH, LDH, and cell count in a cohort of pleural effusions including both transudates and exudates, suggesting that it could aid in adjunct with laboratory measurements in the discrimination of the cause of a pleural effusion [8]. The present study was conducted with the aim of examining potential correlation between the HI and markers of inflammation of PPE as well as clinical parameters of PPE patients.

## 2. Materials and Methods

The study population consisted of patients hospitalized for a PPE at the Respiratory Medicine Department of the University Hospital of Larissa. PPE diagnosis was defined as accumulation of pleural fluid in the setting of an adjacent pneumonia. In order to be included in the study, each patient had a clinical history of febrile illness and purulent sputum. PPE was categorized according to size as follows: small when pleural effusion was recognized by TUS in only one intercostal space, moderate when pleural effusion was recognized in 2 intercostal spaces, and large when pleural effusion was recognized in more than 2 intercostal spaces. Complicated PPE was defined as a parapneumonic pleural effusion with one or more of the following characteristics: pH < 7.2 and/or evidence of micro-organism invasion by culture or Gram stain. In the absence of pH measurements, a low glucose level <40 mg/L was used as a criterion. Complicated PPE in most cases requires chest tube insertion and drainage is indicated since it does not resolve with standard medical care. All patients with complicated PPE in our study were subjected to pleural fluid drainage via chest tube.

All patients underwent a chest x-ray at the first day of their admission along with a TUS evaluation of the pleural space. If pleural fluid was present, an ultrasound-guided diagnostic thoracocentesis would be performed. All patients included in the study were subjected to pleural fluid sampling. The initial clinical and radiological findings were documented, along with the fluid's cytology, culture, and basic biochemical analysis results (including glucose, LDH, pH). ADA was also measured routinely.

**Figure 1:**
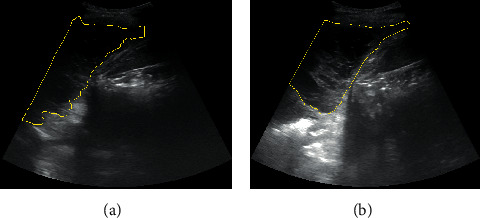
Image cropping technique for analysis using the Image J software. Using the freehand selection, we determine the area of interest. By applying histogram analyses, tissue echo levels are automatically calibrated to the value of 255 for the white pixels and 0 for the black pixels. The mean number of pixels in the selected area is automatically calculated.

A curvilinear probe (2.5–5 MHz) was used for TUS and the ultrasound settings were as follows: dynamic range 60 dBs and depth 8 cm for all the patients. During the initial TUS examination and for better standardization of our method, we used the same anatomic viewpoints for all our ultrasound images. Namely, 5 consequent images were taken to depict the dynamic movement of the fluid during the breathing cycle and five consequent images of the pleural effusion were retrieved through axial view and one from the 10^th^ rib through coronal view and converted into the high-resolution tagged image file format. All images were further processed with a widespread imaging analysis program available for downloading from the public domain (Image J, 1.42q; National Institutes of Health, Bethesda, MD; http://rsb.info.nih.gov/ij). Pleural effusion echogenicity was measured with histogram analysis. Tissue echo levels were automatically calibrated to the value of 255 for the white pixels and 0 for the black pixels. The mean echo levels of all pixels of the pleural effusion and of the 10^th^ rib were counted, and the HI was calculated according to the following formula: HI = mean echo level of all pixels of the rib/mean echo levels of all pixels of pleural effusion, as previously reported [8].  Figure 1 provides a graphic explanation of HI estimation.

Additionally, other qualitative image markers of the parapneumonic effusions were studied via the ultrasound. Pleural effusions were characterized as echogenic or anechoic and the presence or not of loculations was noted, as previously reported [4]. All patients were followed up during their hospitalization and their clinical course was documented along with any medical interventions.

The study was approved by the University Hospital of Larissa ethics committee (ID: 3648–27/05/14). Written and verbal informed consent from all subjects were obtained.

## 3. Statistical Analysis

Data are presented as mean ± standard deviation (SD) for continuous variables or as percentages for categorical variables. Normality was assessed via the one-sample Kolmogorov–Smirnov Test. Relationships of the HI with other variables were assessed with Pearson's *R* or Spearman's correlation coefficients, where appropriate. After choosing the variables highly correlated with HI, a linear regression model was extracted with HI as the dependent variable. For all tests, the level of statistical significance was considered 0.05. The analysis was performed with IBM SPSS 20.0 statistical package (IBM Corporation, San Diego, CA, USA).

## 4. Results

Twenty-four patients (19 males/5 females) with PPE were included in the study. Descriptive data are presented in Table 1 and PPE characteristics are reported in Table 2. Of the patients included, when assessed with TUS, 18 patients presented with echogenic effusion and 6 patients with anechoic effusion. PPEs were also classified based on their size. 8 were small, 8 were moderate, and 8 were large. All PPEs had predominantly polymononuclear cells. Thirteen patients were diagnosed with complicated PPE, while in the remaining 11 patients the pleural infection was resolved with antibiotic treatment and thoracocentesis alone. Two patients died during their hospitalization due to septic shock as a consequence of their severe pleural infection.

Patients with complicated PPE exhibited more days of hospitalization (14.81 ± 9.18 days) and more days to afebrile (7.60 ± 4.73) when compared to patients with uncomplicated PPE (6.12 ± 1.95 and 1.16 ± 0.98, *p*=0.003 and *p*=0.009, respectively, Table 1). As expected, patients with uncomplicated PPE presented lower LDH and higher pH levels than patients with complicated PPE (Table 2). Patients with complicated PPE presented statistically significant increased total cell count, higher polymononuclear cells in pleural fluid, fewer lymphocytes, and higher ADA levels versus patients with uncomplicated PPE (Table 2).

**Figure 2:**
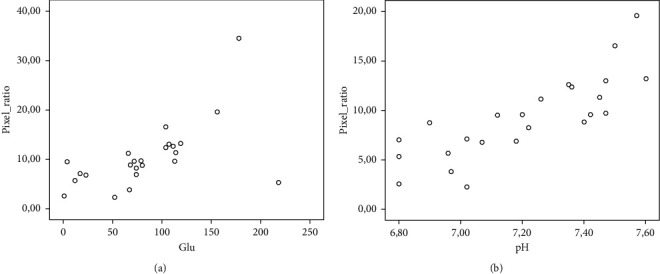
Scatterplot of the predictors (glucose and pH) joining the regression model with the dependent variable (hypoechogenicity index, HI) (*p*=0.022, *r* = 0.474 for glucose, *p* < 0.001, *r* = 0.811 for pH). Pixel ratio stands for HI.

Hypoechogenicity index was lower in patients with complicated PPE when compared to patients with uncomplicated PPE (7.24 ± 2.89 versus 15.39 ± 8.30, *p* < 0.001, Table 2). We observed a statistically significant correlation of the HI with biomarkers that indicate intensity of inflammation such as LDH (*p* < 0.001, *r* = −0.831), glucose (*p*=0.022, *r* = 0.474), and pH (*p* < 0.001, *r* = 0.811) (Figure 2). Moreover, there was a correlation with ADA (*p*=0.005, *r* = −0.552). From the cytological characteristics, there was a statistically significant correlation with total cell number (*p* < 0.001, *r* = −0.657) and polymorphonuclears percentage (*p*=0.02, *r* = −0.590). HI was statistically significantly correlated with days to afebrile (*p*=0.046, *r* = −0.411), duration of chest tube placement (*p* < 0.001, *r* = −0.806), and days of hospitalization (*p*=0.013, *r* = −0.501).

**Figure 3:**
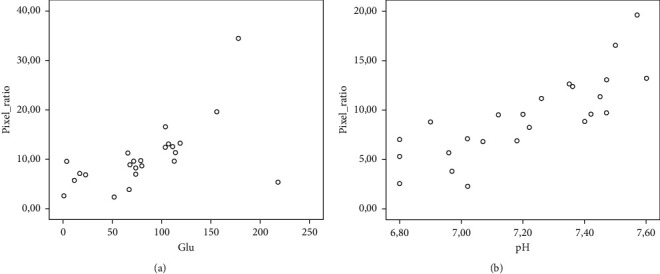
Scatterplot of the predictors (glucose and pH) joining the regression model with the dependent variable (hypoechogenicity index, HI) (*p*=0.022, *r* = 0.474 for glucose, *p* < 0.001, *r* = 0.811 for pH). Pixel ratio stands for HI.

The linear correlation between HI and glucose or pH of the pleural effusion is presented in Figure 3. The linear regression analysis model was used for all the parameters of this study. After testing for a number of methods, the linear model included only the variables pH and glucose, achieving a value of *R*^2^ 53.8%. The coefficient factors and the significance of the proposed model for predicting HI are shown in Table 3.

## 5. Discussion

Our results demonstrated that HI is significantly correlated with the most essential biochemical biomarkers that reflect the intensity of pleural inflammation during the course of parapneumonic effusions such as pH, glucose, and LDH. These biomarkers distinguish complicated from uncomplicated parapneumonic effusions and guide therapeutic decisions such as the need for a chest tube drainage [9, 10]. HI calculation requires less than an minute for every image taken and since our results suggest that HI can effectively reflect PPE intensity of inflammation, one may speculate that HI index may have clinical significance in everyday practice. The associations of HI index with other parameters, such as the increased cell count and the percentage of polymorphonuclear, provide further support to this concept [5, 11, 12].

HI index may reflect the distinct pathophysiological stages of a PPE [13]. The ultrasound image at the first exudative stage of a PPE is usually anechoic or hypoechoic, given the low cell counts and the low content in fibrin. In terms of imaging, this is presented with a low number of pixels with high echogenicity, resulting in higher values of HI index, because those pixels are in the index denominator [13]. Moving to the next stage, there is a bacterial insertion and proliferation in the pleural space, increasing the neutrophil count with a subsequent decrease of pH and glucose values and an increase of LDH values. The fibrin and the septa that are formed gradually following the progression of the inflammation intensity can be reliably detected through ultrasound, either as highly hyperechogenic dots or as thin floating layers, respectively [14]. This leads to a total increase of the echogenic pixels within the effusion and a further decrease of the HI index that could potentially guide PPE treatment. To our knowledge, HI index is the first quantitative correlation index between the conventional ultrasonographic imaging and the biochemical and cytological composition of the pleural fluid in PPE.

Previous studies have shown that chest ultrasound depicts the fibrin and the septa with greater reliability than chest CT [15–17]. In the past, there were several attempts to associate the existence of septa with the differentiation of a complicated or uncomplicated parapneumonic effusion [18–20]. In a recent study, thoracic ultrasound outweighed chest CT and chest x-ray images in the discrimination of complicated PPE with a sensitivity of 69.2% and a specificity of 90% [18]. Most studies are based on the qualitative evaluation of the ultrasonographic image regarding the presence of the septa within the pleural effusion. In our study, the quantitative determination of the HI could potentially outweigh qualitative methods and may define with more accuracy the intensity of the underlying inflammation. The total number of the septa that is included in the quantification of HI is calculated by the number and the echogenicity that the pixels generate while the qualitative recordings are based only on the presence of the septa, without taking into account their number and intensity.

Pleural fluid measurements of pH, glucose, and LDH have been incorporated into medical decision making in daily practice and underline the need for pleural fluid drainage. However, it is documented that encapsulations can frequently occur in some complicated PPEs and especially empyema. Studies have suggested that biochemical markers depend according to the part of the encapsulation that is sampled [21]. pH and glucose values in the aspired fluid may vary so much, which could mislead the physicians in the management of the patient [21]. In such cases, chest ultrasound can depict in detail the possibility of separate spaces in the pleural space. In the determination of HI, the operator can create with accuracy the outline of the image and therefore include the total of the pixels that a multi-encapsulated pleural effusion could generate. The combination of the TUS and HI with biochemical and cytological indicators of the pleural fluid may establish better and faster therapeutic options for the patients with complicated PPE [13].

The relationship of other biomarkers of pleural fluid with the HI index was also assessed in our study, revealing an interesting association of the HI levels with ADA values in patients with PPE. This result is consistent with evidence from other studies, supporting the correlation of the increasing ADA values in the progression of an uncomplicated to complicated PPE possibly due to high-intensity pleural inflammation [11, 22].

One of the most critical findings of our study was the association of HI values with some clinical prognostic markers of PPE. More specifically, HI values were found to be statistically significantly correlated with the duration of the chest tube drainage, the days until apyrexia, and the total length of hospitalization of the patients with PPE that were enrolled in this study, providing further support to the clinical significance of HI. The HI index reflects the hyperechogenic elements of the pleural fluid (such as fibrin, septa, and cell count) and may be used in the prediction of the smooth drainage of a complicated PPE. Researchers have suggested that there is a correlation between septa existence in the TUS image and drainage failure of an underlying PPE [23–25]. HI index provides a quantitative method of PPE inflammation intensity that is performed both easily and fast. Delays in the decision regarding the chest tube insertion of a complicated PPE could lead to the prolongation of days with high temperature, while the procedure of the insertion itself can be related to iatrogenic complications (subcutaneous emphysema, intercostal vascular damage), which may prolong the days of the patient's hospitalization and result in long-term complications such as trapped lung [26, 27].

Our study has several limitations. We acknowledge that our study sample is rather small and larger cohorts are needed to validate our findings. Our study is prospective; however, patients were not treated according to HI values. Future randomized controlled trials that will examine prospectively the safety and effectiveness of HI to guide clinical and therapeutic decisions of patients with PPE are warranted before any definite conclusions are drawn. Additionally, we do not have available ultrasound data from the first days of infection and initiation of symptoms. We acknowledge that the insertion of a chest tube in patients with complicated PPE may affect correlations of HI with days of hospitalization or days to afebrile.

In conclusion, this study provides evidence that HI, a simple, fast, and quantitative ultrasonographic method, may indicate the inflammation intensity of PPE since it correlates with biochemical and microbiological assessments of pleural fluid. HI may be a useful adjunct not only for the follow-up of PPE, but also as a prognostic marker of the clinical course of PPE. More studies are needed in order to investigate further this quantitative method.

## Figures and Tables

**Table 1:** Demographic and clinical characteristics of the study population. Data are expressed as mean ± SD or as absolute numbers. PPE, parapneumonic effusion; M/F, male/female; NA, not applicable; NS, not significant.

Parameter	Uncomplicated PPE	Complicated PPE	*p* value
Subjects number	11	13	NA
Age (years)	65.71 ± 21.18	50.18 ± 15.56	NS
Type of effusion (transudate/exudate)	0/11	0/13	NS
Duration of chest tube placement (days)	0	8.67 ± 10.39	<0.001
Days to afebrile	1.16 ± 0.98	7.60 ± 4.73	0.009
Days of hospitalization	6.12 ± 1.95	14.81 ± 9.18	0.003

**Table 2:** Pleural fluid characteristics of the study population. Data are expressed as mean ± SD or as absolute numbers. PPE, parapneumonic effusion.

Parameter	PPE	Uncomplicated PPE	Complicated PPE	*p* value
Effusion pH	7.20 ± 0.256	7.45 ± 0.77	7.10 ± 0.10	<0.001
Effusion total protein (g/dl)	4.64 ± 0.84	4.35 ± 1.01	4.77 ± 0.76	NS
Effusion albumin (g/dl)	2.45 ± 0.563	2.40 ± 0.55	2.47 ± 0.58	NS
Effusion LDH (IU/L)	2092.33 ± 3503.37	313.62 ± 181.37	2984.43 ± 4031.95	<0.001
Total cell count	12971.25 ± 31649.57	1561.25 ± 1151.79	18648.75 ± 37844.24	0.003
Polymorphonuclears (%)	73.50 ± 18.79	55.85 ± 22.55	82.37 ± 7.23	0.002
Lymphocytes (%)	23.38 ± 17.73	38.37 ± 22.70	15.62 ± 6.84	0.002
ADA(IU/L)	22.46 ± 13.84	11.11 ± 3.90	37.62 ± 32.56	0.001
Hypoechogenicity index	9.22 ± 4.14	15.39 ± 8.30	7.24 ± 2.89	<0.001

**Table 3:** Coefficient factors and the significance of the proposed model for predicting hypoechogenicity index (HI).

Variable	*R* ^2^	*p* value
pH	0.512	0.005
Glucose	0.352	0.043

## Data Availability

The data supporting the conclusions of the present study are presented within the article. The detailed clinical data are not publicly available in order to ensure study subjects anonymity and protect confidentiality. Data are available upon request.
